# On the Complexity of the *Saccharomyces bayanus* Taxon: Hybridization and Potential Hybrid Speciation

**DOI:** 10.1371/journal.pone.0093729

**Published:** 2014-04-04

**Authors:** Laura Pérez-Través, Christian A. Lopes, Amparo Querol, Eladio Barrio

**Affiliations:** 1 Departamento de Biotecnología, Instituto de Agroquímica y Tecnología de Alimentos (CSIC), València, Spain; 2 Grupo de Biodiversidad y Biotecnología de Levaduras, Instituto Multidisciplinario de Investigación y Desarrollo de la Patagonia Norte (IDEPA), CONICET-UNCo, Departmento Química, Facultad de Ingeniería, Universidad Nacional del Comahue, Buenos Aires, Neuquén, Argentina; 3 Departament de Genètica, Universitat de València, València, Spain; University of Strasbourg, France

## Abstract

Although the genus *Saccharomyces* has been thoroughly studied, some species in the genus has not yet been accurately resolved; an example is *S. bayanus*, a taxon that includes genetically diverse lineages of pure and hybrid strains. This diversity makes the assignation and classification of strains belonging to this species unclear and controversial. They have been subdivided by some authors into two varieties (*bayanus* and *uvarum*), which have been raised to the species level by others. In this work, we evaluate the complexity of 46 different strains included in the *S. bayanus* taxon by means of PCR-RFLP analysis and by sequencing of 34 gene regions and one mitochondrial gene. Using the sequence data, and based on the *S. bayanus var. bayanus* reference strain NBRC 1948, a hypothetical pure *S. bayanus* was reconstructed for these genes that showed alleles with similarity values lower than 97% with the *S. bayanus* var. *uvarum* strain CBS 7001, and of 99–100% with the non *S*. *cerevisiae* portion in *S. pastorianus* Weihenstephan 34/70 and with the new species *S. eubayanus*. Among the *S. bayanus* strains under study, different levels of homozygosity, hybridization and introgression were found; however, no pure *S. bayanus* var. *bayanus* strain was identified. These *S. bayanus* hybrids can be classified into two types: homozygous (type I) and heterozygous hybrids (type II), indicating that they have been originated by different hybridization processes. Therefore, a putative evolutionary scenario involving two different hybridization events between a *S. bayanus* var. *uvarum* and unknown European *S. eubayanus*-like strains can be postulated to explain the genomic diversity observed in our *S. bayanus var. bayanus* strains.

## Introduction

The genus *Saccharomyces*, used worldwide to produce different fermented foods and beverages, encompasses the industrially most exploited species known to man. The complex diversity of the genus *Saccharomyces*, including pure, hybrid and introgressed strains, makes species definition difficult and classification controversial. According to the most recent edition of ‘The Yeast, a taxonomic study’ [1], the genus *Saccharomyces* is composed of eight species: *S. arboricolus, S. bayanus*, *S. cariocanus*, *S. cerevisiae*, *S. kudriavzevii*, *S. mikatae*, *S. paradoxus* and *S. pastorianus*. Although several studies have shown that *S. pastorianus* comprises a group of alloploid hybrid strains originated from *S. cerevisiae* and a cryotolerant species similar to *S. bayanus* [2], [3], the last systematic revision maintained the species status for *S. pastorianus* [1].

In a recent study, Libkind et al. [4] isolated and characterized a new *Saccharomyces* species, named *S. eubayanus* and associated with *Nothofagus* spp. trees in Patagonia (Argentina). As the draft genome sequence of this species was closely related to the non *S. cerevisiae* portion of *S. pastorianus* (average divergence of 0.44%), the authors proposed *S. eubayanus* as the previously mentioned *S. bayanus*-like donor of this subgenome in *S. pastorianus* hybrids.

The other controversial *Saccharomyces* taxon is the species *S. bayanus* [1]. *S. bayanus* encompasses a group of cryotolerant strains with active fructose transport, including the former species *S. abuliensis*, *S. bayanus*, *S. globosus*, *S. heterogenicus*, *S. intermedius*, *S. inusitatus*, *S. tubiformis*, *S. uvarum* and *S. willianus*. Based on the quite diverse physiological [5] and genetic [6], [7] traits found among different *S. bayanus* strains, some authors have proposed dividing this taxon into two different species, *S. bayanus* and *S. uvarum* [8], [9]. However, the partial reproductive isolation between the strains of both groups has alternatively suggested the subdivision of the species into two varieties, *bayanus* and *uvarum* [10], which was maintained in the most recent taxonomical review of the genus *Saccharomyces* [1].

Rainieri *et al*. [11] evaluated the genetic variability of 35 yeast strains identified as *S. bayanus* or *S. pastorianus*, and observed a very complex picture. By means of PCR-RFLP and sequencing, the authors confirmed that the type strain of *S. bayanus* (CBS 380^T^) was composed of clearly differentiated ‘*bayanus*’ and ‘*uvarum*’ subgenomes. The authors identified four different genomic compositions among the studied strains: (i) a pure line named *S. uvarum* that included strains containing a single type of genome, with similar physiological and genetic characteristics to the type strain of the former species *S. abuliensis* CBS 7001; (ii) a pure line with a single type of genome named *S. bayanus* that included only strain NBRC 1948; (iii) a hybrid line including strains with portions of the genomes from the two pure lines, as well as alleles termed ‘Lager’ (representing a third genome present in lager brewing strains); iv) a group of *S. cerevisiae*/*S. bayanus*/Lager and *S. cerevisiae*/*S. bayanus*/*S. uvarum*/Lager hybrid strains (*S. pastorianus*). While the pure nature of strain CBS 7001 was confirmed by Libkind et al. [4], these authors together with Nguyen et al. [12] demonstrated that strain NBRC 1948 harbors a mosaic genome composed of a hybrid genetic background belonging to *S. uvarum* and a second unidentified species, which Nguyen et al. provisionally named *S. lagerae*. However, Libkind et al. identified it as belonging to the new species *S. eubayanus*, as well as some small introgressed regions from *S. cerevisiae*.

The main goal of the present study was to decipher the complexity of the *S. bayanus* taxon by performing PCR-RFLP analyses of 34 nuclear genes and by sequencing both nuclear and mitochondrial genes from the 46 different strains identified originally as *S. bayanus* or *S. uvarum*, including the type strains of the former species and the natural isolates from different sources (cider, wine, fruit fermentations, etc.) in the light of the discovery of the new taxon *S. eubayanus*. For this purpose, some *S. pastorianus* strains were also evaluated for comparative purposes. The putative hybridization events responsible for the genomic complexity found in the *S. bayanus* taxon are proposed and discussed.

## Materials and Methods

### Yeasts strains and media

The yeast strains used in this study, together with their sources of isolation and geographical origins, are listed in Table 1. Strains were grown on YPD (w/v: 1% of yeast extract, 2% peptone, 2% glucose) at 28°C and were maintained on YPD supplemented with 2% w/v agar.

**Table 1:** List of *Saccharomyces* strains analyzed in the present study.

Strain reference		Original epithet	Isolation source	Geographic origin	Present characterization
CECT	Other				
1189	CBS 6308		Ale beer	Yorkshire (England)	*S. uvarum*
1369			Unknown	Spain	*S. uvarum*
1884		*S. uvarum*	Wine fermentation	Mentrida (Spain)	*S. uvarum*
1941			Unknown		*S. eubayanus x S. uvarum*
1969^T^	CBS 395^T^	Type of *S.uvarum*	Juice of *Ribes nigrum*	Netherlands	*S. uvarum*
1991	DSMZ 70411		Turbid bottled beer		*S. eubayanus x S. uvarum*
10618			Alpechin	Spain	*S. uvarum*
10174			Unknown	Spain	*S. uvarum*
11035^T^	CBS 380^T^	Type of *S. bayanus*	Beer		*S. eubayanus x S. uvarum*
11036	CBS 381	Type of *S. willianus*	Spoiled beer		*S. uvarum*
11135	CBS375		Unknown		*S. eubayanus x S. uvarum*
11185	NBRC 1948	*S. bayanus*	Unknown		*S. eubayanus x S. uvarum*
11186	NCYC 115		Unknown		*S. eubayanus x S. uvarum*
12600		*S. ellipsoideus*	Sweet wine	Alicante (Spain)	*S. uvarum*
12627		*S. bailli*	Wine	Valladolid (Spain)	*S. uvarum*
12629		*S. uvarum*	Must	Zaragoza (Spain)	*S. uvarum*
12638		*S. uvarum*	Must	León (Spain)	*S. uvarum*
12669		*S. pastorianus*	Grapes	La Rioja (Spain)	*S. uvarum*
12922		*S. carlsbergensis*	Jerez grapes wine	Valladolid (Spain)	*S. uvarum*
12930		*S. bayanus*	Wine	Spain	*S. uvarum*
	CBS 377	Type of *S. intermedius*	Pear wine	Germany	*S. uvarum*
	CBS 378		Unknown	Unknown	*S. eubayanus x S. uvarum*
	CBS 424	Type of *S. globosus*	Pear juice	Meggen (Switzerland)	*S. eubayanus x S. uvarum*
	CBS 425	Type of *S. heterogenicus*	Fermenting apple juice	Tägerwilen (Switzerland)	*S. eubayanus x S. uvarum*
	CBS 431	Type of *S. tubiformis*	Fermenting pear juice		*S. uvarum*
	CBS 1546	Type of *S. inusitatus*	Beer	Rotterdam (Netherlands)	*S. eubayanus x S. uvarum*
	CBS 2898		Wine starter	Herrliberg (Switzerland)	*S. uvarum*
	CBS 2946		Unknown	Unknown	*S. uvarum*
	CBS 2986		Wine	Salenegg (Switzerland)	*S. uvarum*
	CBS 3008		Must of soft fruit	Unknown	*S. eubayanus x S. uvarum*
	NCAIM676		Fermented drink	Hungary	*S. eubayanus x S. uvarum*
	NCAIM677		Fermented drink	Hungary	*S. eubayanus x S. uvarum*
	NCAIM789		*Carpinus betulus* exudate	Babat (Hungary)	*S. uvarum*
	NCAIM868		Slimy material on a stump	Dorog (Hungary)	*S. uvarum*
	S4		Cider	Clonmel (Ireland)	*S. uvarum*
	S10		Cider	Clonmel (Ireland)	*S. uvarum*
	S14		Cider	Clonmel (Ireland)	*S. uvarum*
	S20		Cider	Clonmel (Ireland)	*S. uvarum*
	ZIM 2113		Must of Kraljevina	Dolenjska (Slovenia)	*S. uvarum*
	ZIM 2122		Must of Žametna črnina	Dolenjska (Slovenia)	*S. uvarum*
1940^NT^	CBS 1538^NT^	Neotype of *S. pastorianus*	Lager beer	Denmark	*S. eubayanus* x *S. uvarum*
1970	CBS 1503	Type of *S. monacensis*	Lager beer	Denmark	*S. cerevisiae* x *S. eubayanus*
11037	CBS 1513	Type of *S. carlsbergensis*	Lager beer	Denmark	*S. cerevisiae* x *S. eubayanus*
11000	NCYC 2340		Lager beer	Unknown	*S. cerevisiae* x *S. eubayanus*
1885		*S. cerevisiae*	Wine	Valladolid (Spain)	*S. cerevisiae* x *S. eubayanus*
	S6U		Wine	Italy	*S. cerevisiae* x *S. uvarum*

NT , neotype,

T , type.

Culture collection abbreviated as follows: CECT, Colección Española de Cultivos Tipo, Valencia, Spain; CBS, Centraalbureau voor Schimmelcultures, Utrecht, The Netherlands; DSMZ, German Collection of Microorganisms and Cell Cultures, Braunschweig, Germany; NBRC; NCAIM, National Collection of Agricultural and Industrial Microorganisms, Faculty of Food Sciences, Corvinus University of Budapest, Hungary; NCYC, National Collection of Yeast Cultures, Norwich, UK; ZIM, ZIM Culture Collection of Industrial Microorganisms, University of Ljubljana, Slovenia.

### PCR amplification

The characterization of *S. bayanus* var. *bayanus, S. bayanus* var. *uvarum, S. cerevisiae* and *S. pastorianus* strains was performed by PCR amplification and the subsequent restriction analyses of 34 protein-coding genes distributed along the 16 chromosomes present in these yeasts (Figure S1 in File S1). These genes were probed to be suitable to differentiate among the species of Saccharomyces genus [13]. The oligonucleotides used as primers for the PCR amplifications are provided in Table S1 in File S1.

Although the *S. bayanus var*. *bayanus* and *S. bayanus* var. *uvarum* genomes are almost co-linear to that of *S. cerevisiae*, they differ in several reciprocal translocations, hence some gene regions are located in other linkage groups (Figure S1 in File S1). In this way, *S. bayanus* var. *bayanus* differs from *S. cerevisiae* in two reciprocal translocations among chromosomes II and IV and VIII [3], [14], while *S. bayanus* var. *uvarum* contains two other translocations between chromosomes VI and X, and between XIV and IItIV [15].

Total yeast DNA was isolated following standard procedures [16]. PCR reactions were performed in a final volume of 100 μl containing 10 μl of 10x *Taq* DNA polymerase buffer, 100 μM deoxynucleotides, 1 μM of each primer, 2 units of *Taq* DNA polymerase (BioTools, B&M Labs, Madrid, Spain) and 4 μl of DNA diluted to 1–50 ng/μl. PCR amplifications were carried out in Techgene and Touchgene thermocyclers (Techne, Cambridge, UK) as follows: initial denaturing at 95°C for 5 min, then 40 PCR cycles involving the following steps: denaturing at 95°C for 1 min, annealing at 55°C (for most genes), and extension at 72°C for 2 min, then a final extension at 72°C for 10 min. For genes *ATF1*, *DAL1*, *EGT2*, *KIN82*, *MNT2*, *MRC1*, *RRI2* and *UBP7*, annealing was performed at 50°C.

PCR products were run on 1.4% agarose (Pronadisa, Madrid, Spain) gels in 0.5x TBE buffer. After electrophoresis, gels were stained with 0.5 μg/ml of ethidium bromide solution (AppliChem, Darmstadt, Germany) and were visualized under UV light. A 100-bp DNA ladder marker (Roche Molecular Biochemicals, Mannheim, Germany) served as a size standard.

### Restriction analysis of nuclear gene regions

Simple digestions with different endonuclases were performed with 15 μl of amplified DNA to a final volume of 20 μl. Restriction endonucleases *Acc* I, *Asp* I, *Asp* 700I, *Cfo I, Dde* I, *Eco* RI, *Hae* III, *Hin*d III, *Hin*f I, *Msp* I, *Pst* I, *Rsa* I, *Sac* I, *Scr* FI, *Taq* I *and Xba* I (Roche Molecular Biochemicals, Mannheim, Germany) were used according to the supplier's instructions. Restriction fragments were separated on 3% agarose (Pronadisa, Madrid, Spain) gel in 0.5 x TBE buffer. A mixture of 50-bp and 100-bp DNA ladder markers (Roche Molecular Biochemicals, Mannheim, Germany) served as size standards.

### Amplification, cloning, sequencing and phylogenetic analysis of nuclear genes

The 34 gene regions used in this study were amplified and sequenced in NBRC 1948 strain for the g*enetic reconstruction of a hypothetical S. bayanus var. bayanus* genome. For genes *EPL1*, *GSY1*, *JIP5*, *KIN82*, *MRC1, PEX2, MAG*2, *NPR2* and *ORC1* additional sequences were obtained (sequences obtained from strains CECT 11186 and CBS 424). Additionally, new alleles were sequenced to confirm their nature (“*uvarum*”, “*eubayanus*” or “*cerevisiae*” alleles). These sequences were deposited in the nucleotide databases under accession numbers KJ093508 to KJ093569.

For gene *MNL1*, no diagnostic restriction patterns for the differentiation of the ‘*eubayanus*’- and ‘*uvarum*’-type alleles were found. Therefore, the *MNL1* PCR products were sequenced, in all the strains, for allele discrimination, and the corresponding sequences were deposited under accession numbers KJ093570 to KJ093618

The PCR products were purified using the Perfectprep Gel Cleanup Kit (Eppendorf, Hamburg, Germany) following the manufacturer's instructions, and were subsequently sequenced for allele discrimination. Sequencing was performed with the BigDye Terminator V3.1 Cycle Sequencing Kit (Applied Biosystems, Warrington, UK) according to the manufacturer's instructions. The sequencing reactions were run on a Techgene Thermal Cycler (Techne, Cambridge, UK), which was programmed as follows: an initial denaturation at 94°C for 3 min, followed by 99 cycles of denaturation at 96°C for 10 s, annealing at 50°C for 5 s, and polymerization at 60°C for 4 min. Sequences were obtained with an Applied Biosystems automatic sequencer model ABI 3730 (Applied Byosistems, Warrington, UK).

For the heterozygous strains exhibiting ambiguous nucleotide sequences, given the presence of more than one allele, the PCR amplifications were cloned and sequenced to obtain the nucleotide sequence of each allele. Cloning was carried out with the pGEM T Easy Vector System ll kit (Promega, Madison, USA) by preparing a ligation reaction with a final volume at 3.3 μL and by incubating overnight at 4°C. The transformation reaction was performed with 20 μL of competent cells JM 109 (Promega, Madison, USA) and 2 μL of the ligation reaction, and the mix was incubated by shaking at 200 rpm for 1.5 h. A volume of 120 μL was plated in LB medium (1% tryptone, 0.5% yeast extract, 1% glucose, 1.5% agar) with 100 μg/mL ampicillin, 0.5 mM IPTG, and 80 μg/mL X-Gal. Plates were incubated for 24 h at 37°C and at least 12 positive colonies were isolated for the direct PCR amplification from colony, and the subsequent sequencing was done according to the conditions described above.

Alignments were done using the Clustal W algorithm as implemented in the MEGA 4.0 software [17]. Similarities between ‘*eubayanus*’ and ‘*uvarum*’ alleles were estimated as nucleotide identities per 100 sites (%).

The jModelTest program [18] was used to estimate the evolutionary model that best represents the nucleotide divergence data provided by the *MNL1* sequences by applying the Bayesian information criterion [19]. The best fitting model was the Kimura 2-paremetter model [20] with a gamma distribution (G) of substitution rates with a shape parameter of α = 0.099. A maximum likelihood (ML) tree was obtained with PHYML 3.0 [21] by applying the corresponding K2-p +G model. The statistical support for the resulting topology was assessed using a nonparametric bootstrap with 100 pseudo-replicates [22].

### Amplification, sequencing and phylogenetic analysis of COX2

To establish the *COX2* gene haplotypes present in the strains under study, this mitochondrial gene region was PCR-amplified and subsequently sequenced given the absence of diagnostic restriction sites. *COX2* was amplified using the primers and conditions described in Belloch *et al.* [23]. PCR products were cleaned with the Perfectprep Gel Cleanup kit (Eppendorf, Hamburg, Germany) and both DNA strands were sequenced directly using the BigDyeTM Terminator v3.0 Cycle Sequencing kit (Applied Biosystems, Warrington, UK) following the manufacturer's instructions, in an Applied Biosystems automatic DNA sequencer Model ABI 3730.

*COX2* sequences (accesion numbers AF442212, AJ938046, AJ938045, AJ966729, and JN676768 to JN676813) were aligned and analyzed with the MEGA 4 program [17]. Due to low divergences and the presence of a putative recombination, phylogenetic trees were obtained by the Neighbor-Joining method using the p-distance (uncorrected nucleotide divergence). Tree reliability was assessed using a nonparametric bootstrap with 2000 pseudo-replicates.

## Results

### Genetic reconstruction of a hypothetical S. bayanus var. bayanus or ‘bayanus’ pure line

By using a set of the 34 pairs of primers (Table S1 in File S1) previously generated in our laboratory for *Saccharomyces* hybrids detection and characterization, the nuclear gene regions of NBRC 1948 strain were amplified and sequenced. This strain was selected as the most representative *S. bayanus* var. *bayanus* strain, because was defined by Rainieri et al. [11] as a “pure” *S. bayanus* var. *bayanus* strain. These sequences were then compared to the homologous regions of the genome sequence of strain CBS 7001(available at http://www.saccharomycessensustricto.org). This strain, also known as MCYC623, is considered as a pure *S. bayanus* var. *uvarum* strain [11].

Each pair of homologous sequences were aligned and the corresponding nucleotide similarities were estimated as shown in Table 2. From the 34 gene compared regions, identical sequence pairs for genes *EPL1*, *GSY1*, *JIP5*, *KIN82*, *MRC1* and *PEX2* (100% similarity), and almost identical sequences for genes *MAG*2, *NPR2* and *ORC1* (99.6 to 99.9% similarity), were observed. However, 25 homologous sequence pairs showed similarities lower than 97%, and between 86.0% for *CBP*2 and 96.7% for *MET6* (Table 2).

**Table 2:** Sequence similarity for 34 protein-coding genes between the reference strain of *S. bayanus var. bayanus* (NBRC 1948) and the reference strains of *S. bayanus* var. *uvarum* (CBS 7001) and the ‘*eubayanus*’ alleles of the *S. pastorianus* strain Weihenstephan 34/70 (W 34/70).

Gene	Similarity (%) between CBS 7001 and			Similarity (%) between W 34/70 ‘*eubayanus*’ alleles and			Similarity (%) between CBS 7001 and W 34/70 ‘*eubayanus*’ alleles
	NBRC 1948	CECT 11186	CBS 424	NBRC 1948	CECT 11186	CBS 424	
*APM3*	92.7	-	-	100	-	-	92.7
*ATF1*	91.2	-	-	99.2	-	-	91.8
*BAS1*	91.9	-	-	100	-	-	91.9
*BRE5*	86.7	-	-	100	-	-	86.7
*BUD14*	92.1	-	-	99.9	-	-	92.0
*CAT8*	91.9	-	-	99.6	-	-	92.1
*CBP2*	86.0	-	-	100	-	-	86.0
*CBT1*	91.4	-	-	99.4	-	-	91.6
*CYC3*	91.5	-	-	100	-	-	91.5
*CYR1*	93.2	-	-	100	-	-	93.2
*DAL1*	92.0	-	-	99.9	-	-	92.2
*EGT2*	88.6	-	-	99.7	-	-	88.9
*EPL1*	**100**	92.6	92.6	92.7	99.9	99.9	92.5
*EUG1*	90.3	-	-	99.5	-	-	90.4
*GAL4*	91.2	-	-	none	-	-	none
*GSY1*	**100**	95.4	95.4	95.4	99.7	99.7	95.4
*JIP5*	**100**	**100**	91.9	91.9	91.9	96.6	91.9
*KEL2*	87.7	-	-	99.9	-	-	87.8
*KIN82*	**100**	92.3	**99.7**	none	None	none	none
*MAG2*	**99.9**	**99.9**	93.9	94.0	94.0	100	93.9
*MET6*	96.7	-	-	100	-	-	96.7
*MNL1*	89.6	-	-	100	-	-	89.6
*MNT2*	91.0	-	-	100	-	-	91.0
*MRC1*	**100**	90.7	**99.8**	90.7	99.2	90.7	90.7
*NPR2*	**99.7**	93.0	93.0	93.2	99.9	99.9	92.9
*OPY1*	92.8	-	-	100	-	-	92.8
*ORC1*	**99.6**	89.5	**99.7**	89.7	100	89.7	89.5
*PEX2*	**100**	**100**	92.4	92.3	92.3	99.9	92.3
*PKC1*	91.9	-	-	100	-	-	91.9
*PPR1*	95.6	-	-	99.6	-	-	95.8
*RPN4*	90.5	-	-	100	-	-	90.5
*RRI2*	90.1	-	-	100	-	-	90.1
*UBP7*	92.5	-	-	100	-	-	92.5
*UGA3*	91.0	-	-	99.9	-	-	91.3

For those genes exhibited between the CBS 7001 and NBRC 1948 similarities ≥99.6% (in bold), additional sequences were obtained for *S. bayanus* strains CECT 11186 or CBS 424, exhibiting divergent alleles, and their similarities to CBS 7001 and W 34/70 are also provided. Those gene sequence comparisons for which *S. pastorianus* Weihenstephan 34/70 contains only ‘*cerevisiae*’ alleles are indicated by ‘none’ (i.e., no *eubayanus* alleles are present to compare).

To check if the nine identical or almost identical sequences found in both NBRC 1948 and CBS 7001 could be fixed characteristics of the *S. bayanus* species genome, sequences for those genes were obtained from two other *S. bayanus* var. *bayanus* strains, CECT 11186 (NCYC 115) and CBS 424. Three sequences from CECT 11186 (*JIP5*, *MAG2* and *PEX2*) and three others from CBS 424 (*KIN82*, *MRC1* and *ORC1*) were identical or almost identical to the sequences in CBS 7001 and NBRC 1948. However, all the remaining sequences analyzed in the two additional strains gave lower similarity values, around 89.5–95.4%, as compared to the reference sequences (Table 2).

By combining the sequence data from strains NBRC 1948, CECT 11186 and CBS 424, a complete set of ‘*bayanus*’ alleles of a hypothetical *S. bayanus* var. *bayanus* pure line (alleles with similarity values lower than 97% as compared to strain CBS 7001) was obtained.

Using the sequences obtained in this study, we performed a genome BLAST search on the non *cerevisiae* sub-genome of the *S. pastorianus* strain Weihenstephan 30/70 available in NCBI. Two homologous sequences were obtained for all genes, each corresponding to one of the two subgenomes (the *S. eubayanus* and *S. cerevisiae* subgenomes according to Libkind et al. [4]). The only exceptions were genes *KIN82* and *GAL4*, for which only one highly similar sequence to *S. cerevisiae* was obtained. Sixteen gene sequences from the ‘*eubayanus*’ fraction of *S. pastorianus* were 100% identical to the sequences comprising our hypothetical *S. bayanus* pure line, and 15 gene sequences were almost identical (between 99.2% and 99.9% of similarity)(Table 2).

The divergent genes between the ‘*uvarum*’ pure line CBS 7001 and our hypothetical ‘*bayanus*’ pure line (the alleles from NBRC 1948, CECT 11186 or CBS 424) were also divergent between CBS 7001 and the ‘*eubayanus*’ Weihenstephan 34/70 gene sequences (similarities of 86.0–96.7%) (Table 2).

After considering the high similarity of the gene sequences between the ‘*eubayanus*’ alleles in the *S. pastorianus* strain and our ‘hypothetical *S. bayanus* var. *bayanus*’, we used the name ‘*eubayanus*’, or simply ‘E’, to designate these alleles henceforth. Within this new framework, strain CBS 7001 contained only ‘*uvarum*’ alleles (or simply ‘U’), but strain NBRC 1948 contained both E and an important fraction of U alleles (26.5% of the genes under study). Our results indicate that the two alleles have an average divergence of 8.4% (between 3.3% and 14%) for the analyzed gene sequences.

### Characterization of the strains belonging to the *S. bayanus* taxon based on the presence of both alleles ‘eubayanus, E’ and ‘uvarum, U’

To characterize the complex *S. bayanus* taxon and to find a putative pure *S. bayanus* var. *bayanus* strain, a PCR-RFLP analysis of the 34 gene regions was performed on a panel of 46 strains deposited in culture collections under species name *S. bayanus, S. uvarum* or *S. pastorianus* (Table 1). According to the sequence differences observed between alleles E and U, only the restriction endonucleases able to differentiate both alleles for each particular gene were chosen from those proposed by González et al.[13] (Table S2 in File S1). New restriction endonucleases were used for gene sequences for which the enzymes proposed by González et al [13] did not differentiate between the two alleles (Table S2 in File S1). In order to merely avoid wrong allele type assignation due to intra-type sequence variations, we used a single restrictase to assign U or E alleles only when more than two restriction site gains/losses were observed between both alleles (because small variant of the alleles can sometimes make one fragment get cutted into 2 fragments while the rest of the pattern remains the same). Whenever this condition was not achieved with a single restrictase, additional restriction enzymes were used.

**Figure 1:**
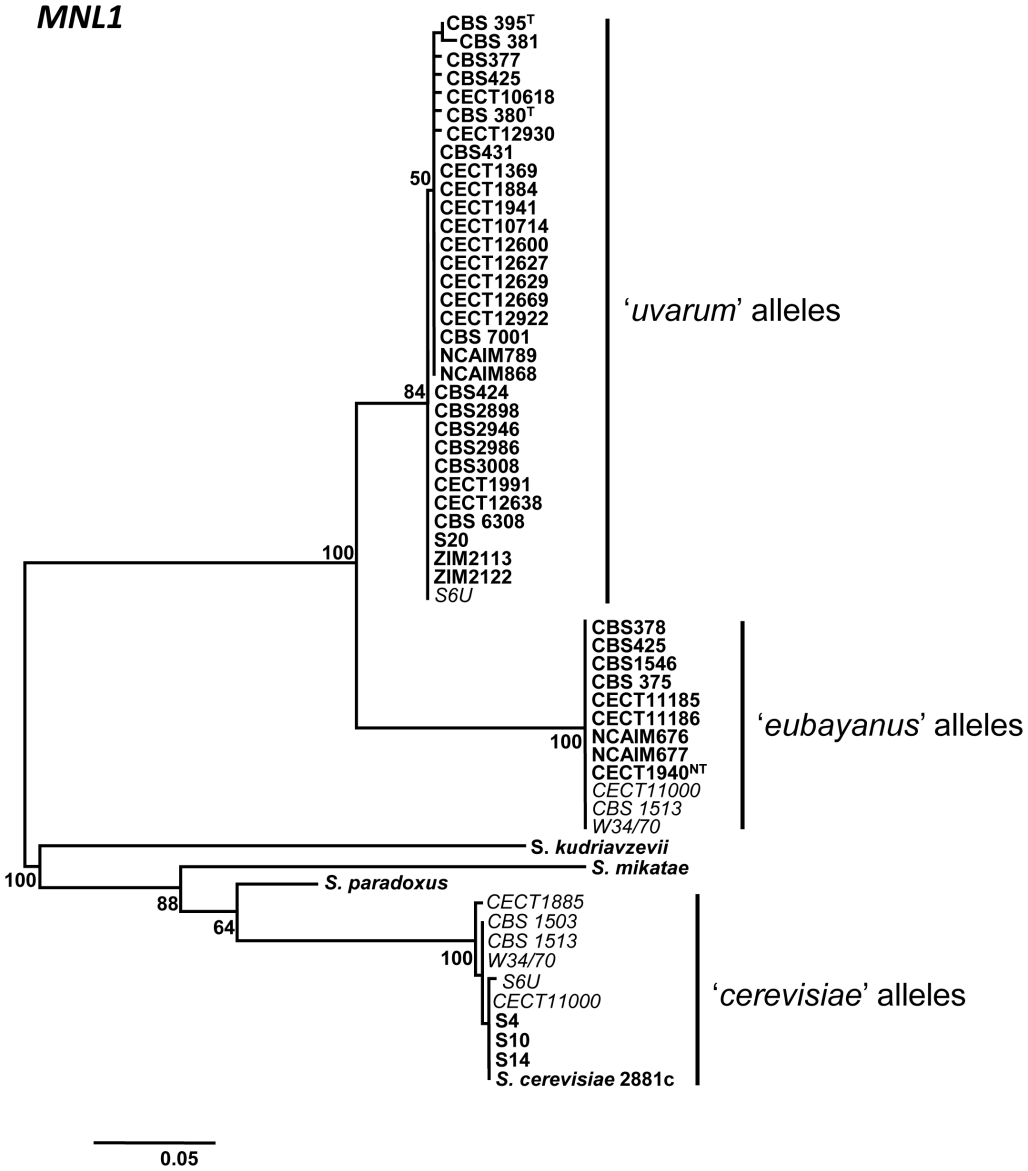
Phylogenetic tree obtained with the partial sequences of the nuclear *MNL1* gene. Numbers at nodes correspond to the bootstrap values based on 1000 pseudo-replicates. The scale is in nucleotide substitutions per site. All the strains are indicated in bold, except the *S. pastorianus* ones.

Accordingly, the restriction patterns similar to those present in reference strain *S. bayanus* var. *uvarum* CBS 7001 were named ‘U1’, while those present in the reconstructed pure *S. bayanus* var. *bayanus* and in *S. pastorianus* strain Weihenstephan 34/70 (from *S. eubayanus*) were named ‘E1’. The restriction patterns similar to those present in reference strain *S. cerevisiae* S288c were named ‘C1’. As we were unable to find diagnostic restriction patterns to differentiate alleles E and U for the *MNL1* gene region, the analysis of the *MNL1* region was done by sequencing (Figure 1).

**Figure 2:**
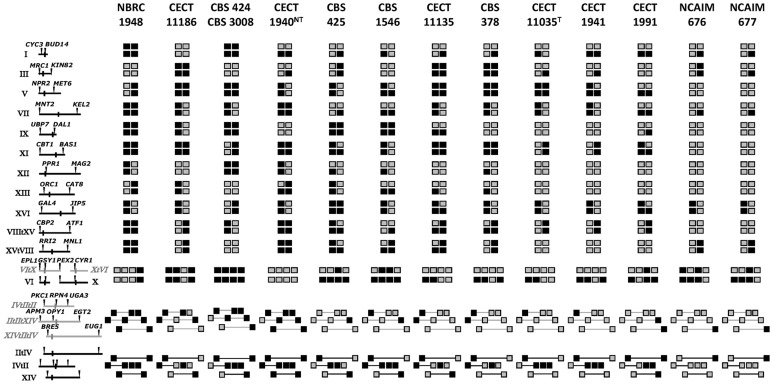
RFLPs of 34 nuclear genes from the *S. bayanus* strains analyzed in this work. Each square corresponds to a copy of each gene region according to its chromosome location, indicated in the map on the left. ‘*eubayanus*’ alleles are indicated as black squares and ‘*uvarum*’ alleles as gray squares. The genes order differs between *S. bayanus* var. *bayanus* and *S. bayanus* var. *uvarum*, as depicted, due to the presence of two translocations. The first involves chromosomes VI and X in *S. bayanus* var. *bayanus* and chromosomes VItX and XtVI in *S. bayanus* var. *uvarum*, while the second involves chromosomes IItIV, IVtII and XIV in *S. bayanus* var. *bayanus* and chromosomes IItIItIV, IVtIItII and XIVtIItIV in *S. bayanus* var. *uvarum*.

Following the procedures described before, we obtained a complete characterization of all the strains listed in Table 1. These results are summarized in Figure 2 and Tables S4 and S5 in File S1. Some strains exhibited alternative restriction patterns, which differed by one restriction site gains/losses from the C1, E1 or U1 patterns present in the reference strains. These new alleles were sequenced and their similarities with the reference C, U and E alleles were tested. These new alleles were named C, E or U (depending on the closest allele), followed by an ordinal number from 2 onward, as shown in Table S3 in File S1.

Twenty-seven of the 46 strains showed only U alleles for 33 of the 34 analyzed nuclear gene sequences. Seven of them showed U alleles for the 34 analyzed genes and twenty showed a C2 allele for *PEX2* gene region, being the most frequent alternative allele detected among the analyzed strains (20 of the 27 strains bearing only U alleles showed this C2 allele). Among them, 13 different nuclear genotypes were observed due to the presence of alternative U2 alleles for different gene regions (Table S4 in File S1). These new allele variants were observed only for genes *MNT2*, *UBP7*, *BAS1*, *RRI2* and *BRE5*. Most of these strains exhibited only one allele for the 34 analyzed genes, except for strains ZIM 2122 and NCAIM 868, which were heterozygous U1/U2 for genes *RRI2* and *BAS1*, respectively. Finally, Irish cider strains S4, S10, and S14 contained a similar combination of alleles U1 and U2 to that found in strains CBS 2946 and NCAIM 789 for all genes analyzed, except for gene *MNL1* (Table S4 in File S1), for which they showed a ‘*cerevisiae*’ (C) allele, as observed after the sequence analysis (Figure 1).

Fourteen strains contained different combinations of alleles U and E (Table S5 in File S1), indicating their ‘*uvarum*’ x ‘*eubayanus*’ hybrid nature. These included strains NBRC 1948, the type strain of *S. bayanus* CBS 380^T^ and the putative neotype strain of *S. pastorianus* CECT 1940^NT^. It was possible to clearly differentiate these U x E hybrids into two groups (types I and II) according to their genetic constitution. To obtain a more illustrative picture of this situation, we represent the genetic constitution of these strains containing alleles U and E in Figure 2. The strains included in Type I (strains NBRC 1948, CECT 11186, CBS 424 and CBS 3008) appeared to be homozygous for all 34 genes under study (Figure 2); while the alloploid strains that presented some genes in heterozygosis (U/E alleles) were included in Type II (Figure 2 and Table S5 in File S1). The total number of heterozygous E/U loci varied from 9% in strain NCAIM 676 to 44% in strain CBS 1546. Alternative E2 alleles were observed for genes *DAL1* (strains CBS 424 and CBS 3008) and *BAS1* (strains CBS 424, CBS 3008, CBS 425 and CECT 1991), while alternative U2 alleles were observed for genes *MNT2*, *UBP7*, *BAS1*, *RRI1* and *BRE5* in different strains (Table S5 in File S1).

Another group of strains included those identified as *S. pastorianus* and were, therefore, characterized by the additional presence of ‘*cerevisiae*’ (C) alleles (Table S6 in File S1). Among them, wine commercial strain S6U exhibited alleles U and C for 33 genes and alleles C1 and C2 for *PEX2* gene. Three strains, including the former type strains of *S. carlsbergensis* (CBS 1513 = CECT 11037) and *S. monacensis* (CBS 1503 = CECT 1970) and one wine strain (CECT 1885), contained different combinations of alleles E and C. Finally, lager brewing strain CECT 11000 contained the three types of alleles (E, C and U), although alleles U were found for only five gene regions, including *MRC1*, *NPR2*, *KEL2*, *GSY1* and *EGT2* (Table S6 in File S1). Interestingly, all the previously mentioned yeasts (except S6U) exhibited alleles E2 for two genes: *BAS1* and *BRE5*.

According to our data, no pure strains bearing 100% E alleles were found among our *S. bayanus* strains. Based on the presence of alleles E and U, it was possible to divide the *S. bayanus* strains analyzed in this work into three groups: (i) a ‘*S. bayanus* var. *uvarum*’ pure-line group that includes those strains containing only U, in which some limited *S. cerevisiae* introgressions may have occurred, as with strains S04, S10 and S14, showing a C allele in the subtelomeric gene *MNL1* or the 20 strains showing a C2 allele in the subtelomeric gene *PEX2*; (ii) a homozygous ‘*S. bayanus* var. *bayanus*’ group including strains with both alleles E and U in homozygosis (Type I); (iii) an alloploid ‘*S. bayanus* var. *bayanus*’ group containing strains with both alleles E and U in heterozygosis (Type II).

It was also possible to divide the *S. pastorianus* strains into three groups: (i) hybrids with alleles C, E and U (ii) hybrids with alleles C and E and (iii) hybrids with alleles C and U.

### About the origin of mitochondrial DNA in *S. bayanus*

**Figure 3:**
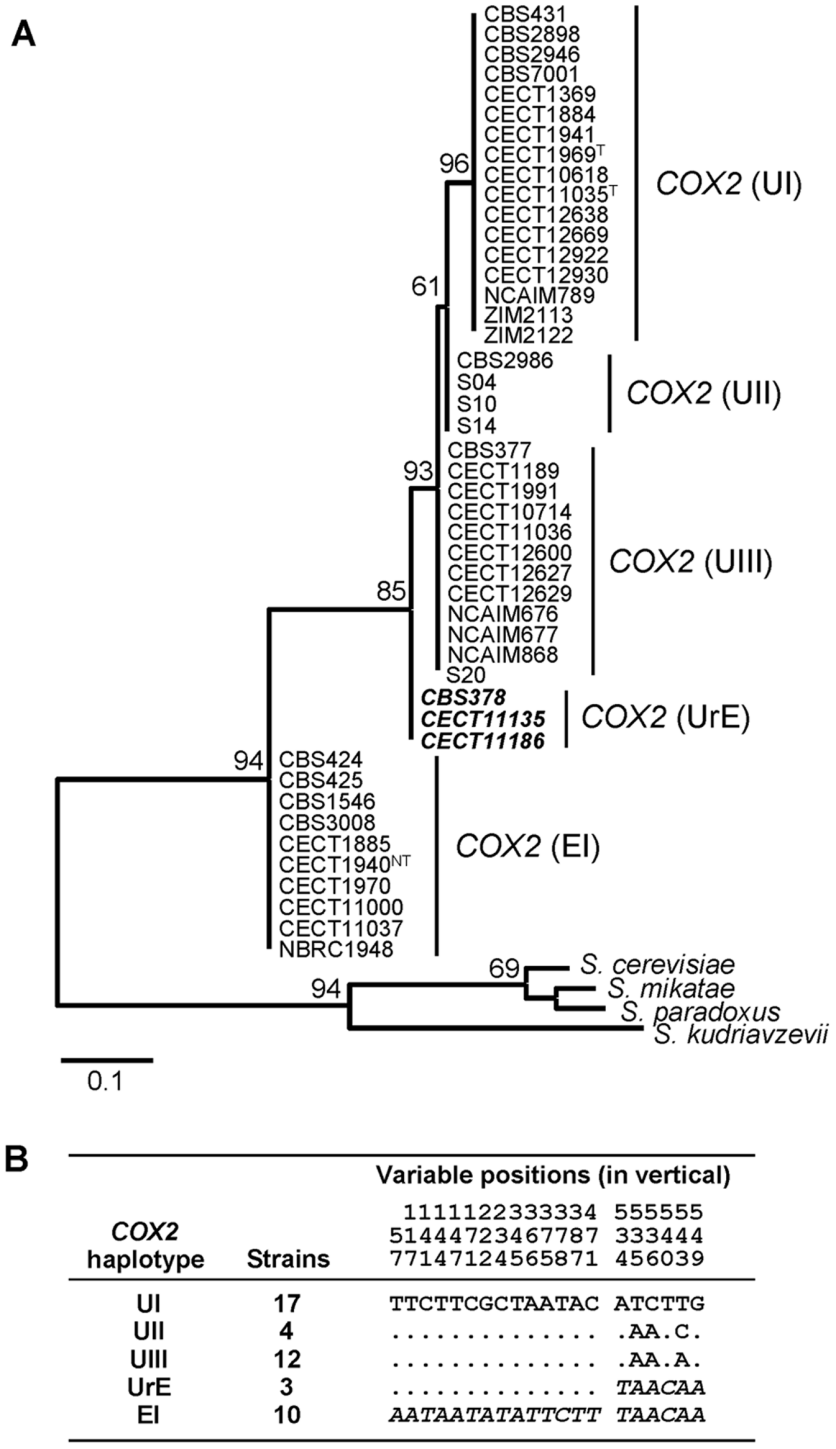
Phylogenetic analysis of the partial sequences of the mitochondrial *COX2* gene. **A- NJ tree**. Numbers at nodes correspond to the bootstrap values based on 1000 pseudo-replicates. The scale is in nucleotide substitutions per site. Strains with a controversial adscription are indicated in bold. **B- Variable positions on the different COX2 haplotypes**. A dot indicates the presence of the same nucleotide at this position. Haplotype U-I is used as a reference.

In order to obtain a more complete picture of the identity of the *S. bayanus* strains studied, we also analyzed the nature of their mtDNA. For this purpose, we evaluated mitochondrial gene *COX2* from all 46 strains. Due to the difficulties in unveiling *COX2* variability in *Saccharomyces* by restriction analyses, we performed direct sequencing. Five groups of strains were separated according to the *COX2* phylogenetic analysis (Figure 3A). The strains possessing only U haplotypes for the 34 analyzed genes (the ‘*uvarum*’ pure line strains) were separated into three *COX2* haplotypes: U-I, U-II and U-III. Haplotype U-I, found in reference strain CBS 7001, was the most frequent among our strains. Haplotype U-II was shared by cider strains and wine strain CBS2986, and haplotype U-III was observed in nine strains of diverse origins (Figure 3A).

All the *S. pastorianus* strains CBS 1503, CBS 1513, CECT 1885 and CECT 11000 showed the same haplotype E–I, postulated as being received from the *S*. *eubayanus* progenitor according to the phylogenetic analysis of the sequences. The only exception was strain S6U, which exhibited an *S. cerevisiae COX2* haplotype (Figure 3A).

*S. bayanus* hybrids strains, with alleles U and E in their nuclear genes, exhibited four different *COX2* haplotypes which did not cluster together in the gene phylogeny. Some of their *COX2* sequences clustered with ‘*uvarum*’ haplotypes U–I, U-II and U-III, and others did so with the ‘*eubayanus*’ haplotype E-I present in *S. pastorianus* strains (Figure 3A). Interestingly, three *S*. *bayanus* hybrid strains, CECT 11186, CBS 375 and CBS 378, exhibited a *COX2* haplotype located in the phylogenetic tree at an intermediate position between the *uvarum* and *eubayanus* allele (E–I). A detailed analysis of the variable positions of the *COX2* sequences (Figure 3B) showed that the 5′region of this haplotype was identical to the ‘*uvarum*’ haplotype sequences, but differed from the ‘*eubayanus*’ sequence, while the 3′region was identical to the ‘*eubayanus*’ sequence and differed from the ‘*uvarum*’. This result are indicative that these three hybrid strains may exhibit a putative recombinant *COX2* haplotype (called UrE), which could result from the recombination between the *uvarum* and *eubayanus COX2* genes. To check this putative recombination, we performed separate phylogenetic analyses of the 5′ and 3′ *COX2* regions (Figure S2 in File S1) corresponding to nucleotide positions 1 to 525 and nucleotides 526 to 582, respectively. Accordingly in the 5′region phylogeny, the UrE haplotype clustered with the *uvarum* haplogroup and with the *eubayanus* E-I allele in the 3′ region phylogeny.

## Discussion

Most studies about complex species “*S. bayanus*” coincide on the existence of two well-differentiated groups of strains: the molecularly and physiologically heterogeneous group of strains belonging to *S. bayanus* var. *bayanus*, and the homogenous group of strains pertaining to *S. bayanus* var. *uvarum* [1]. These two varieties have even been considered to be two different species (*S. bayanus* and *S. uvarum*, respectively) by other authors because of their partial reproductive isolation [8], [9]. However, the genetically heterogeneous nature of the ‘*bayanus*’ variety, as several works have demonstrated [4], [11], [12], makes it difficult to obtain reliable information about hybridization data to evaluate the reproductive isolation between these two varieties. Together with the discovery of the pure species *S. eubayanus* and the association of this new taxon with the ‘*bayanus*-like’ subgenome of *S. pastorianus*, Libkind et al. [4] proposed the use of *S. eubayanus* and *S. uvarum* as descriptors of species, but restricted the name *S. bayanus* to the hybrid lineages between pure species. *S. eubayanus* has not been detected in Europe; however, in order to explain its necessary contact with a *S. cerevisiae* ale strain to generate the hybrid *S. pastorianus*, it is feasible that this species inhabits a specific niche environment still to be sampled in this continent, as suggested by Gibson *et al.* [24].

For the purpose of finding a European strain of *S. eubayanus*, a set of 46 European strains obtained from different sources and annotated as *S. bayanus* in different culture collections have been genetically characterized. It is interesting to note that most analyzed strains (∼85%) were diploid (preliminary results not shown). As expected, most of the gene alleles found in the *S. bayanus* var. *bayanus* reference strain NBRC 1948 were divergent (6–8% of nucleotide divergence) as compared to the same ones in the *S. bayanus* var. *uvarum* reference strain CBS 7001. These divergence values were similar to those found between the pure lines of *S. eubayanus* and *S. bayanus* var. *uvarum* [4]. Contrarily, a significant fraction of identical or almost identical alleles was found between NBRC 1948 and CBS 7001 (27% of the genes under study). In a similar study, but with 35 *S. bayanus* and *S. pastorianus* strains (only nine strains coincide with our study), Rainieri et al., [11] have also identified alleles with high similarity between strains NBRC 1948 and CBS 7001. In their study, the authors considered that those alleles correspond to cases in which both the ‘*bayanus*’ and ‘*uvarum*’ varieties show same or similar allelic variants. In our work, these kinds of identical or almost identical alleles between the two varieties are considered ‘*uvarum*’ (U) due to the divergence found between the genes common to NBRC 1948 and CBS 7001 and the non *S. cerevisiae* portion in *S. pastorianus* Weihenstephan 34/70. Accordingly, these genes also evidence the non-pure nature of strain NBRC 1948. According to our results, the alleles named ‘*bayanus*’ by Rainieri et al.[11], which differed from ‘*uvarum*’ alleles in only a few nucleotidic positions, must be reconsidered to be ‘*uvarum*’ variants. Following the same argument, the ‘lager’ alleles in Rainieri et al. [11] must correspond to the real ‘*bayanus*’ alleles because they demonstrate a homology percentage of around 89–94% between these *lager* and ‘*uvarum*’ alleles, which are similar results to those observed in the present work between ‘*uvarum*’ and ‘eu*bayanus*’ alleles.

The reconstructed *S. bayanus* var. *bayanus* pure line, which contains a combination of alleles present in different hybrid *S. bayanus* strains, shows a similarity of 99–100% with the non *S. cerevisiae* subgenome of the fully sequenced *S. pastorianus* lager strain Weihenstephan 34/70. After considering the genetic similarity demonstrated between *S. eubayanus* and the non *S. cerevisiae* portion of *S. pastorianus* [4], and as no complete database containing the whole *S. eubayanus* genome exists, we assigned the name ‘*eubayanus*’ instead of ‘*bayanus*’ to the non *uvarum* alleles in the *S. bayanus var. bayanus* strains (*S. eubayanus* x *S. uvarum* hybrids) analyzed in our work. Following the idea proposed by Gibson et al. [24], this hypothetical genotype may represent the genotype exhibited by a European pure line of *S. eubayanus*.

Of the 46 strains analyzed, 7 only exhibited U alleles for the 34 analyzed gene regions, 17 exhibited U alleles for 33 gene regions and a C allele for *PEX2*, and 3 exhibited U alleles for 32 gene regions and C alleles for *PEX2* and *MNL1*, and hence, they can be considered pure *S. bayanus* var. *uvarum* or *S. uvarum* strains. These strains were isolated mainly from grapes, grape must or wine, but also from pear or apple ciders, while a few were isolated from other sources; i.e., spoiled and ale beers, *alpechin* (olive mill waste), or tree exudates. Low variation in allele composition was observed among these strains in the *S*. *uvarum* group. This intraspecific homogeneity has also been evidenced in recent studies using microsatellite loci analyses [25], [26]. Nevertheless, the presence of heterozygous strains in this group can be considered evidence for a certain degree of interbreeding among the strains of this variety. The sequence analysis of mitochondrial gene COX2 is also in accordance with this homogeneity, which was detected in the nuclear DNA for all the S. uvarum strains. *COX2* is a highly variable gene that has proved most informative in determining the interspecies phylogenetic relationships in the *Saccharomyces*-*Kluyveromyces* complex [23], [27] and different interspecific hybrids of the genus *Saccharomyces* [13], [28], [29].

Twenty strains from the *S. uvarum* group, isolated from Irish cider, wine, beer, as well as different unfermented musts and natural environments, exhibited a *S*. *cerevisiae* introgression in gene *PEX2*, located in a subtelomeric region of the translocated *S. uvarum* chromosome VItX; 3 of them, isolated from Irish cider, presented a second introgression in gene *MNL1*, also located in a subtelomeric region of the translocated *S. uvarum* chromosome XVtVIII. The presence of *S. cerevisiae* subtelomeric sequences has been previously reported for the *S. bayanus var. bayanus* [9], [30] and *S. bayanus var. uvarum* [25] strains. According to the above-cited authors, the *S. cerevisiae* sequences in the *S. bayanus* genomes are the result of introgression following unstable interspecies hybridization [31], [32]. Introgression may be particularly effective for regaining lost traits, which were functional in a common ancestor; in other words, introgression often serves as a repair or replacement strategy [33]. The two introgressed strains identified by Naumova *et al.* [25] as *S. bayanus* var. *uvarum* are also included in the present study where we demonstrate that they contain ‘*eubayanus*’ alleles in nine different gene regions; hence they must be reclassified as hybrid *S. bayanus* var. *bayanus* strains. Introgressed *S. cerevisiae* telomeric Y′ sequences have also been described in three cider *S. bayanus* var. *uvarum* strains from Brittany and Normandy, France [34]. As Naumova et al. proposed [25], introgressed *S. bayanus* var. *uvarum* strains could be isolated if both, *S. cerevisiae* and *S. bayanus* var. *uvarum*, strains co-exist in the same environment, allowing hybridization [35], and, according our results, introgressions in subtelomeric regions seem to be quite frequent.

### Origin of the *S. bayanus* var. *bayanus* genome complexity

The situation of the strains classified as *S. bayanus var. bayanus* is more complex due to the presence of different combinations of ‘*uvarum*’ and ‘*eubayanus*’ alleles in their nuclear genomes, as well as mtDNA of different origins, as indicated by the presence of the ‘*uvarum*’ or ‘*eubayanus*’ *COX2* haplotypes, as well as a rare possible recombinant haplotype.

The recombination between mitochondrial DNAs from different parental strains has already been described for *S. cerevisiae* in early studies into yeast mitochondrial genetics [36]. In *S. cerevisiae*, mitochondria from the two parental spores can fuse in the zygote after mating. In these fused mitochondria, parental mtDNAs mix and recombine to generate a recombinant lineage that can be established as homoplasmic during mitochondrial vegetative segregation [36]. *COX2* recombination was also postulated to occur in natural *S. cerevisiae* x *S. kudriavzevii* hybrids [37], and in this study, we showed that it could also have occurred in *S. bayanus* hybrids.

**Figure 4:**
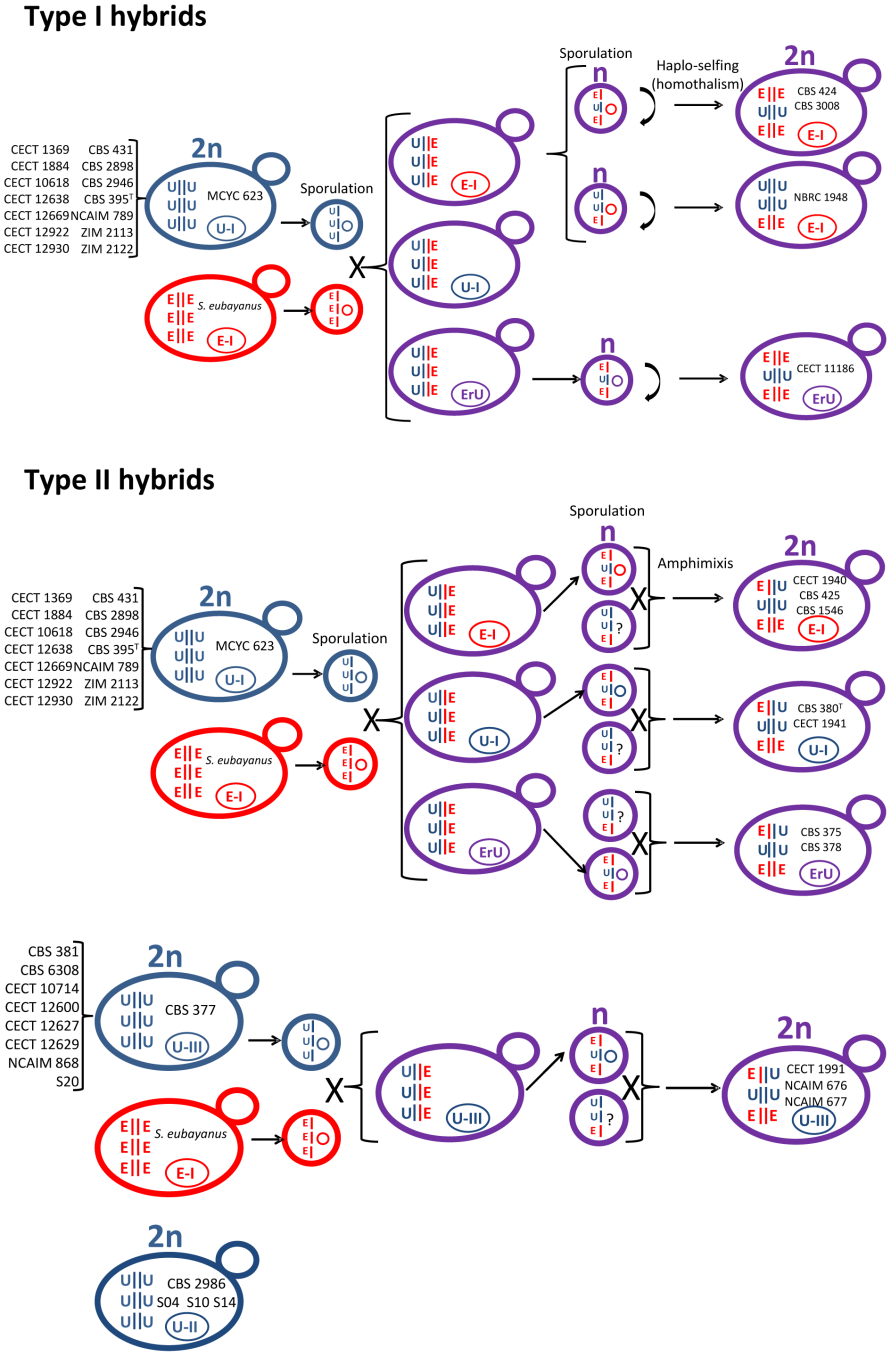
Possible origins of the *S. bayanus* (*S. uvarum x S. eubayanus*) hybrid strains.

Based on the complexity observed in nuclear and mtDNA genes, we propose a scheme (Figure 4) to summarize the generation of all the different S. *bayanus* var. *bayanus* strains resulting from several hybridization events between pure strains from the ‘*uvarum*’ group and a strain possessing only ‘*eubayanus*’ alleles, which is related to the recently described Patagonian *S*. *eubayanus*, and is also similar to a European *S. eubayanus* strain.

Based on the fact that two different ‘*uvarum*’ *COX2* alleles were detected among the strains in the ‘*bayanus*’ hybrid group, we hypothesized that at least two ‘*uvarum*’ pure strains were involved in the origin of the complete set of *S. bayanus* strains studied here. Regardless of the parental ‘*uvarum*’ involved in hybridization, the first generation of hybrids would possess a complete set of chromosomes from each parental species, and should show reduced fertility according to the low spore viability (7%) exhibited by the artificial hybrids between *S. eubayanus* and *S. uvarum* generated by Libkind et al. [4]. In addition, Liti et al. [38] had shown that a sequence divergence of over 5% between strains considerably reduces spore viability, and the average genome divergence between *S. eubayanus* and *S. uvarum* is ∼6.9% [4].

After hybridization, and perhaps in a fermenting environment context where these hybrids are found and can reach extremely large yeast populations, some of these hybrids can sporulate to generate some viable spores (<7%) that can give rise to the two hybrid types I and II (Figure 4) described in this work. Type I hybrids can arise through sporulation and haplo-selfing (homothallism), while Type II hybrids do so by sporulation and amphimixis, or by mating those spores carrying different a genetic background (heterothallism) (Figure 4). In this ancestral hybrid, recombination between “*uvarum*” and “*eubayanus*” chromosomes was also possible during sporulation.

Hybrid speciation has been performed under laboratory conditions from artificial *S. cerevisiae* x *S. paradoxus* hybrids [39] and, despite being uncommon, it has been described in plants [40]. This is partly due to the ability to self-fertilize, which produces identical homolog pairs for every chromosome (except at the mating-type locus on chromosome III), thus avoiding any incompatibility that might arise by fusion with other gametes, even from the same parent [39].

Although the initial hybrids obtained from *S. eubayanus* and *S. uvarum* mating show low spore viability [4], derived type I hybrids would probably recover a higher fertility due to their homozygosity. Contrarily, type II hybrids bearing an important proportion of genes in heterozygosis (E–U) should show lower fertility than the type I hybrids. According to this hypothesis, the larger number of heterozygous genes in hybrids, the lower their fertility. In this sense, spore viability for some of these strains was evaluated by Naumov [10]. In that study, Naumov observed 48% and 7% of spore viabilities for strains CBS 380 and CBS 425, respectively, corresponding to heterozygous type II strains in the present study, and 77% of spore viability for CBS 424, a homozygous type I strain.

It is important to note that, with the exception of the work by Libkind et al. [4], all previous studies evaluated the viability of the hybrids generated by mating a *S. bayanus* var. *uvarum* pure strain (i.e., CBS 7001) and different *S. bayanus* var. *bayanus* strains, such as NBRC 1948 or CBS 380, which correspond according to this study to *S. bayanus* hybrid lines. In this context, the analysis of hybrid fertility is confusing and extremely variable because they really correspond to backcrosses between hybrids and representative strains of the parental species, while the fertility of the progeny depends on the genome constitution of the hybrid.

### Saccharomyces pastorianus

As previously mentioned, the most popular examples of hybrid yeasts are those involved in lager beer production included in the taxa *S. pastorianus* (syn. *S. carlsbergensis* and *S. monacensis*), which originated from natural hybridization between *S. cerevisiae* and *S. eubayanus* [4]. Although the different *S. pastorianus* strains possess chromosomes from both species [41], [42], mitochondrial DNA was always acquired from the non *cerevisiae* parental [43]. Recent comparative genome hybridization studies have shown that *S. pastorianus* strains came about as a result of at least two independent hybridization events [12], [44]. These hybridizations generated the two main groups of lager brewing strains, including Frohberg-type lager strains (i.e., Weihenstephan 34/70) and Saaz-type lager strains (i.e., *S. monacensis* CBS 1503 = CECT 1970).

Our study also agrees partly with these data as Saaz-type lager strains CECT 1885, CECT 11000, CBS 1503 (the type strain of *S. monacensis*) and CBS 1513 (the type strain of *S. carlsbergensis*) exhibit an E2 fixed allele for genes *BRE5* and *BAS1*, whilst strain Weihenstephan 34/70 (a Frohberg-type lager strain) possesses an E1 allele for the same gene regions.

No ‘*cerevisiae*’ alleles were detected in strain CECT 1940, considered the type strain of *S. pastorianus*. This fact indicates that two different strains can be found under the same name, CECT 1940. The literature reports this confusion as to the use of the neotype of *S. pastorianus* several times, and it has been attributed to the misuse of two of its neotype strains: CBS1538^NT^ (from the CBS) and NRRLY-1551 (from the ARS collection in the past). A recent study by Nguyen et al. [12], together with the proteomic data reported by Joubert et al. [45], demonstrate that NRRLY-1551 has been misidentified and should be reclassified as *S. bayanus*. Our results coincide with the data reported by Nguyen et al [12], but we also observed that the strain CECT 1940 used in this work (probably originating from strain NRRLY-1551, and not from CBS 1538) is a *S. bayanus* hybrid strain that bears both the ‘*uvarum*’ and ‘*eubayanus*’ alleles.

Finally, while strain S6U seems to have been originated from the hybridization of a *S. bayanus* var. *uvarum* pure strain (such as CBS 381, CBS 431 and CECT 1884) and a *S. cerevisiae*, the remaining *S. pastorianus* strains evaluated in this work were obtained from the hybridization between a *S. eubayanus* strain (strains Weihenstephan 34/70, CECT 1885, CBS 1503 and CBS 1513) or *S. bayanus* (bearing both ‘*eubayanus*’ and ‘*uvarum*’ alleles; i.e., CECT 11000) and a *S. cerevisiae* strain. The fact that wine strain *S. pastorianus* CECT 1885 shows ‘*eubayanus*’ alleles does not fall in line with the hypothesis of Naumova et al. [30], which suggests that the non ‘*cerevisiae*’ parental of *S. pastorianus* wine strains is always a *S. bayanus var. uvarum*, unless this strain is a contaminant strain from brewing environments.

In this work, the complexity of “*S. bayanus*” species group was deciphered using the restriction analysis of 34 genes used to differentiate ‘uvarum’ (U) and ‘eubayanus’ (E) alleles. From the 48 analyzed strains none was a pure *S. bayanus var. bayanus/S. eubayanus* strain. The ‘*uvarum*’ group showed a high intraspecific homogeneity, although a certain degree of interbreeding among the strains of this variety was shown. The situation of the ‘*bayanus*’ group is more complex, all these stains are hybrids between *S. uvarum* and *S. eubayanus* and can be divided in two subgroups: type I or homozygous strains and type II or heterozygous strains. A scheme summarizing the generation of all the different S. *bayanus* var. *bayanus* is proposed.

## Supporting Information

File S1 **Contains the files: Figure S1 Chromosome composition and gene order in different Saccharomyces species.** **A**- *S. cerevisiae*. **B**- *S. eubayanus*. **C**- *S. uvarum*. **Figure S2 Phylogenetic analysis of the 5′ and 3′ regions of the mitochondrial** ***COX2*** **gene.** **A**- 5′ region. **B**- 3′ region. **Table S1 Gene regions under restriction analysis and primers used for PCR amplification.** Chromosome (Chr) positions of the genes correspond to S. cerevisiae, for other arrangements present in the other strains see Figure S1. **Table S2 Composite restriction patterns deduced from the gene region sequences of the** ***eubayanus*** **-type alleles, present in the reference strains** ***S. bayanus*** **NBRC 1948, CECT 11186, CBS 424 or** ***S. pastorianus*** **Weihenstephan 34/70, the** ***uvarum*** **alleles exhibited by** ***S. uvarum*** **CBS 7001, and the** ***cerevisiae*** **-type alleles present in** ***S. cerevisiae*** **S288c.** These composite patterns for each gene region have been named after the initial of the allele-type name followed by the order numeral 1. Chromosome (Chr) positions of the genes correspond to *S. cerevisiae*, for other arrangements present in the other strains see Figure S1. **Table S3 Alternative restriction patterns exhibited by** ***S. bayanus*** **or** ***S. uvarum*** **strains differing by one or two restriction site gains/losses (indicated in bold) from those found in the reference strains**. **Table S4 Conformation of the** ***S. uvarum*** **strains for each gene region according to the composite restriction patterns exhibited.** For a description of the composite restriction patterns, see Tables S2 and S3. Mitochondrial *COX2* sequence haplotypes are described in Figure 2. **Table S5 Conformation of the** ***S. bayanus*** **strains with** ***eubayanus*** **- and** ***uvarum*** **-type alleles according to the composite restriction patterns exhibited.** For a description of the composite restriction patterns, see Tables S2 and S3. Mitochondrial *COX2* sequence haplotypes are described in Figure 2. **Table S6 Conformation of the** ***S. pastorianus*** **strains with** ***eubayanus*** **-** ***cerevisiae*** **- or** ***uvarum*** **-type alleles according to the composite restriction patterns exhibited.** For a description of the composite restriction patterns, see Tables S2 and S3. Mitochondrial *COX2* sequence haplotypes are described in Figure 2, except S6U *COX2*, which is similar to *S. cerevisiae* (C) *COX2*.
(ZIP)
