# Supplementary material for: On the Complexity of the Saccharomyces bayanus Taxon: Hybridization and Potential Hybrid Speciation
Source: PLoS One. 2014 Apr 4;9(4):e93729. doi: 10.1371/journal.pone.0093729 (PMC3976317; doi:10.1371/journal.pone.0093729)
Supplement: File S1 — Contains the files: Figure S1 Chromosome composition and gene order in different Saccharomyces species. A- S. cerevisiae. B- S. eubayanus. C- S. uvarum. Figure S2 Phylogenetic analysis of the 5′ and 3′ regions of the mitochondrial COX2 gene. A- 5′ region. B- 3′ region. Table S1 Gene regions under restriction analysis and primers used for PCR amplification. Chromosome (Chr) positions of the genes correspond to S. cerevisiae, for other arrangements present in the other strains see Figure S1. Table S2 Composite restriction patterns deduced from the gene region sequences of the eubayanus -type alleles, present in the reference strains S. bayanus NBRC 1948, CECT 11186, CBS 424 or S. pastorianus Weihenstephan 34/70, the uvarum alleles exhibited by S. uvarum CBS 7001, and the cerevisiae -type alleles present in S. cerevisiae S288c. These composite patterns for each gene region have been named after the initial of the allele-type name followed by the order numeral 1. Chromosome (Chr) positions of the genes correspond to S. cerevisiae, for other arrangements present in the other strains see Figure S1. Table S3 Alternative restriction patterns exhibited by S. bayanus or S. uvarum strains differing by one or two restriction site gains/losses (indicated in bold) from those found in the reference strains. Table S4 Conformation of the S. uvarum strains for each gene region according to the composite restriction patterns exhibited. For a description of the composite restriction patterns, see Tables S2 and S3. Mitochondrial COX2 sequence haplotypes are described in Figure 2. Table S5 Conformation of the S. bayanus strains with eubayanus - and uvarum -type alleles according to the composite restriction patterns exhibited. For a description of the composite restriction patterns, see Tables S2 and S3. Mitochondrial COX2 sequence haplotypes are described in Figure 2. Table S6 Conformation of the S. pastorianus strains with eubayanus - cerevisiae - or uvarum -type alleles according to t [file pone.0093729.s001.zip › Table S2.docx]

Table S2 Composite restriction patterns deduced from the gene region sequences of the *eubayanus*-type alleles, present in the reference strains *S. bayanus* NBRC 1948, CECT 11186, CBS 424 or *S. pastorianus* Weihenstephan 34/70, the *uvarum* alleles exhibited by *S. uvarum* CBS 7001, and the *cerevisiae-*type alleles present in *S. cerevisiae* S288c. These composite patterns for each gene region have been named after the initial of the allele-type name followed by the order numeral 1. Chromosome (Chr) positions of the genes correspond to *S. cerevisiae*, for other arrangements present in the other strains see Figure 1.

| **Chr** | Gene | **Rest. enzyme** | ***uvarum-*type**  **allele** | **Pat-tern** | ***eubayanus-*type**  **allele** | **Pat-tern** | ***S.cerevisiae***  **S288C** | **Pat-tern** |
| --- | --- | --- | --- | --- | --- | --- | --- | --- |
| **I** | ***CYC3*** | *Cfo* I | 490 30 15 5 | **U1** | 360 170 10 | **E1** | 540 | **C1** |
|  |  | *Hae*III | 330 230 10 |  | 230 180 150 10 |  | 330 200 10 |  |
|  |  | *Hin*f I | 260 175 130 |  | 270 140 100 |  | 175 160 110 90 |  |
|  | ***BUD14*** | *Msp* I | 875 160 130 | **U1** | 560 315 160 130 | **E1** | 475 310 230 95 40 | **C1** |
|  |  | *Taq* I | 340 280 240 210 70 25 |  | 560 340 230 30 |  | 420 300 290 100 55 |  |
| **II** | ***PKC1*** | *Asp* 700 I | 460 440 280 | **U1** | 900 280 | **E1** | 480 470 230 | **C1** |
|  | ***OPY1*** | *Scr*F I | 370 85 | **U1** | 250 120 85 | **E1** | 350 105 | **C1** |
|  |  | *Taq* I | 235 125 95 |  | 220 155 80 |  | 420 300 290 100 55 |  |
|  | ***APM3*** | *Msp* I | 960 70 | **U1** | 690 340 | **E1** | 750 280 | **C1** |
|  |  | *Hin*f I | 375 210 190 175 80 |  | 345 210 190 175 110 |  | 640 220 170 |  |
| **III** | ***MRC1*** | *Dde* I | 590 180 80 75 | **U1** | 590 270 75 | **E1** | 470 240 100 75 40 | **C1** |
|  |  | *Msp* I | 760 170 |  | 930 |  | 720 160 50 |  |
|  | ***KIN82*** | *Hae* III | 430 330 170 | **U1** | 930 | **E1** | 500 430 | **C1** |
|  |  | *Taq* I | 530 350 25 25 |  | 930 |  | 650 280 |  |
| **IV** | ***UGA3*** | *Hin*f I | 260 210 165 140 80 | **U1** | 490 310 55 | **E1** | 305 295 225 | **C1** |
|  |  | *Taq* I | 325 225 175 80 70 |  | 175 150 150 125 125 80 70 |  | 225 220 170 100 80 70 |  |
|  | ***RPN4*** | *Taq* I | 265 240 145 110 75 | **U1** | 325 220 180 110 | **E1** | 725 100 10 | **C1** |
|  | ***EUG1*** | *Hae* III | 500 130 80 60 50 | **U1** | 710 110 | **E1** | 630 130 60 | **C1** |
| **V** | ***NPR2*** | *Hin*f I | 460 190 100 70 50 35 25 | **U1** | 460 245 190 35 | **E1** | 760 110 60 | **C1** |
|  | ***MET6*** | *Asp* I | 680 | **U1** | 340 340 | **E1** | 500 180 | **C1** |
|  |  | *Asp* 700 I | 440 240 |  | 680 |  | 440 240 |  |
| **VI** | ***EPL1*** | *Hae* III | 435 375 320 | **U1** | 1130 | **E1** | 555 435 145 | **C1** |
|  | ***GSY1*** | *Eco*R I | 770 | **U1** | 450 320 | **E1** | 500 270 | **C1** |
|  |  | *Hae* III | 515 230 25 |  | 770 |  | 640 80 50 |  |
| **VII** | ***MNT2*** | *Msp* I | 540 280 150 | **U1** | 540 250 150 50 | **E1** | 835 135 | **C1** |
|  |  | *Scr*F I | 545 210 150 70 |  | 325 220 210 150 70 |  | 635 340 |  |
|  | ***KEL2*** | *Hae* III | 680 360 120 50 | **U1** | 360 350 330 120 50 | **E1** | 1010 120 50 20 10 | **C1** |
|  |  | *Msp* I | 880 330 |  | 670 280 160 100 |  | 780 230 200 |  |
| **VIII** | ***CBP2*** | *Cfo* I | 445 340 | **U1** | 610 175 | **E1** | 370 340 75 | **C1** |
|  |  | *Hin*f I | 370 370 35 |  | 370 255 125 35 |  | 310 290 110 35 |  |
|  | ***MNL1*** | Alleles differentiated by sequencing analysis | | | | | | |
| **IX** | ***UBP7*** | *Hae* III | 740 160 90 | **U1** | 545 445 | **E1** | 990 | **C1** |
|  |  | *Hin*f I | 405 405 165 15 |  | 405 405 165 15 |  | 805 160 15 10 |  |
|  | ***DAL1*** | *Hae* III | 345 170 100 75 65 5 | **U1** | 470 210 80 | **E1** | 286 260 211 5 | **C1** |
| **X** | ***PEX2*** | *Hae* III | 230 215 125 95 45 | **U1** | 270 190 150 60 45 | **E1** | 345 260 110 | **C1** |
|  | ***CYR1*** | *Hin*d III | 395 155 | **U1** | 550 | **E1** | 405 155 | **C1** |
|  |  | *Msp* I | 550 |  | 550 |  | 390 170 |  |
|  |  | *Sac* I | 550 |  | 340 210 |  | 560 |  |
| **XI** | ***CBT1*** | *Hae* III | 300 180 | **U1** | 360 120 | **E1** | 480 | **C1** |
|  |  | *Msp* I | 240 210 30 |  | 430 50 |  | 480 |  |
|  | ***BAS1*** | *Hae* III | 380 370 200 120 | **U1** | 690 380 | **E1** | 800 240 | **C1** |
|  |  | *Msp* I | 930 150 |  | 1080 |  | 1080 |  |
| **XII** | ***PPR1*** | *Taq* I | 260 185 140 70 55 | **U1** | 260 240 140 70 | **E1** | 230 185 165 125 | **C1** |
|  |  | *Xba* I | 465 245 |  | 710 |  | 710 |  |
|  | ***MAG2*** | *Msp* I | 835 175 | **U1** | 600 225 175 | **E1** | 480 400 110 | **C1** |
|  |  | *Taq* I | 455 330 125 60 40 |  | 330 310 290 60 40 |  | 470 375 125 40 |  |
| **XIII** | ***ORC1*** | *Hae* III | 530 350 | **U1** | 430 350 100 | **E1** | 670 180 | **C1** |
|  |  | *Taq* I | 455 215 150 60 |  | 295 240 155 75 60 55 |  | 700 150 |  |
|  | ***CAT8*** | *Msp* I | 360 250 200 | **U1** | 360 330 120 | **E1** | 690 120 | **C1** |
| **XIV** | ***EGT2*** | *Hin*f I | 270 110 55 | **U1** | 325 110 | **E1** | 435 | **C1** |
|  |  | *Scr*F I | 430 5 |  | 240 195 |  | 405 30 |  |
|  | ***BRE5*** | *Hin*f I | 335 300 145 | **U1** | 396 235 95 50 | **E1** | 290 230 100 65 60 40 | **C1** |
| **XV** | ***RRI2*** | *Hin*f I | 515 440 125 60 | **U1** | 515 235 215 165 | **E1** | 565 190 165 125 60 50 | **C1** |
|  |  | *Taq* I | 575 490 40 35 15 |  | 420 300 240 170 20 |  | 585 380 145 45 |  |
|  | ***ATF1*** | *Cfo* I | 560 200 150 | **U1** | 560 350 | **E1** | 560 350 | **C1** |
|  |  | *Hae* III | 435 275 200 |  | 435 235 200 40 |  | 635 235 40 |  |
| **XVI** | ***GAL4*** | *Scr*F I | 410 190 85 75 | **U1** | 760 | **E1** | 430 330 | **C1** |
|  |  | *Taq* I | 310 300 90 45 15 |  | 300 170 140 140 |  | 220 210 180 140 |  |
|  | ***JIP5*** | *Msp* I | 710 | **U1** | 580 130 | **E1** | 455 130 125 | **C1** |
|  |  | *Scr*F I | 710 |  | 450 260 |  | 710 |  |
