# Supplementary material for: On the Complexity of the Saccharomyces bayanus Taxon: Hybridization and Potential Hybrid Speciation
Source: PLoS One. 2014 Apr 4;9(4):e93729. doi: 10.1371/journal.pone.0093729 (PMC3976317; doi:10.1371/journal.pone.0093729)
Supplement: File S1 — Contains the files: Figure S1 Chromosome composition and gene order in different Saccharomyces species. A- S. cerevisiae. B- S. eubayanus. C- S. uvarum. Figure S2 Phylogenetic analysis of the 5′ and 3′ regions of the mitochondrial COX2 gene. A- 5′ region. B- 3′ region. Table S1 Gene regions under restriction analysis and primers used for PCR amplification. Chromosome (Chr) positions of the genes correspond to S. cerevisiae, for other arrangements present in the other strains see Figure S1. Table S2 Composite restriction patterns deduced from the gene region sequences of the eubayanus -type alleles, present in the reference strains S. bayanus NBRC 1948, CECT 11186, CBS 424 or S. pastorianus Weihenstephan 34/70, the uvarum alleles exhibited by S. uvarum CBS 7001, and the cerevisiae -type alleles present in S. cerevisiae S288c. These composite patterns for each gene region have been named after the initial of the allele-type name followed by the order numeral 1. Chromosome (Chr) positions of the genes correspond to S. cerevisiae, for other arrangements present in the other strains see Figure S1. Table S3 Alternative restriction patterns exhibited by S. bayanus or S. uvarum strains differing by one or two restriction site gains/losses (indicated in bold) from those found in the reference strains. Table S4 Conformation of the S. uvarum strains for each gene region according to the composite restriction patterns exhibited. For a description of the composite restriction patterns, see Tables S2 and S3. Mitochondrial COX2 sequence haplotypes are described in Figure 2. Table S5 Conformation of the S. bayanus strains with eubayanus - and uvarum -type alleles according to the composite restriction patterns exhibited. For a description of the composite restriction patterns, see Tables S2 and S3. Mitochondrial COX2 sequence haplotypes are described in Figure 2. Table S6 Conformation of the S. pastorianus strains with eubayanus - cerevisiae - or uvarum -type alleles according to t [file pone.0093729.s001.zip › Table S6.docx]

**Table S6 Conformation of the *S. pastorianus* strains with *eubayanus*- *cerevisiae*- or *uvarum*-type alleles according to the composite restriction patterns exhibited.** For a description of the composite restriction patterns, see Supporting Information Tables S2 and S3. Mitochondrial *COX2* sequence haplotypes are described in Fig 2, except S6U *COX2*, which is similar to *S. cerevisiae* (C) *COX2*.

| ***S. bayanus* Chrom.** | ***S. uvarum* Chrom.** | ***S. cerevisiae* Chrom.** | **Gene** | **W 34/70** | **S6U** |  | **CECT 1885** | **CBS**  **1503** | **CECT 11000** | **CBS**  **1513** |
| --- | --- | --- | --- | --- | --- | --- | --- | --- | --- | --- |
| **mtDNA** | **mtDNA** | **mtDNA** | ***COX2*** | **EI** | **C** |  | **EI** | **EI** | **EI** | **EI** |
| **I** | **I** | **I** | ***CYC3*** | **E1 C1** | **U1 C1** |  | **E1 C1** | **E1 C1** | **C1** | **E1 C1** |
| **I** | **I** | **I** | ***BUD14*** | **E1 C1** | **U1 C1** |  | **E1 C1** | **C1** | **C1** | **C1** |
| **III** | **III** | **III** | ***MRC1*** | **E1 C1** | **U1 C1** |  | **E1 C1** | **E1** | **U1 C1** | **C1** |
| **III** | **III** | **III** | ***KIN82*** | **C1** | **U1 C1** |  | **E1** | **E1** | **C1** | **C1** |
| **V** | **V** | **V** | ***NPR2*** | **E1 C1** | **U1 C1** |  | **E1 C1** | **E1** | **U1 C1** | **E1 C1** |
| **V** | **V** | **V** | ***MET6*** | **E1 C1** | **U1 C1** |  | **E1** | **E1** | **E1 C1** | **E1 C1** |
| **VII** | **VII** | **VII** | ***MNT2*** | **E1 C1** | **U1 C1** |  | **C1** | **C1** | **E1 C1** | **E1 C1** |
| **VII** | **VII** | **VII** | ***KEL2*** | **E1 C1** | **U1 C1** |  | **E1** | **E1** | **U1 C1** | **E1 C1** |
| **IX** | **IX** | **IX** | ***UBP7*** | **E1 C1** | **U1 C1** |  | **E1 C1** | **E1 C1** | **E1 C1** | **E1 C1** |
| **IX** | **IX** | **IX** | ***DAL1*** | **E1 C1** | **U1 C1** |  | **E1 C1** | **E1 C1** | **E1 C1** | **E1 C1** |
| **XI** | **XI** | **XI** | ***CBT1*** | **E1 C1** | **U1 C1** |  | **E1 C1** | **E1 C1** | **E1 C1** | **E1 C1** |
| **XI** | **XI** | **XI** | ***BAS1*** | **E1 C1** | **U1 C1** |  | **E2** | **E2** | **E2 C1** | **E2** |
| **XII** | **XII** | **XII** | ***PPR1*** | **E1 C1** | **U1 C1** |  | **E1** | **E1** | **E1 C1** | **E1** |
| **XII** | **XII** | **XII** | ***MAG2*** | **E1 C1** | **U1 C1** |  | **E1** | **E1** | **E1 C1** | **E1** |
| **XIII** | **XIII** | **XIII** | ***ORC1*** | **E1 C1** | **U1 C1** |  | **E1 C1** | **E1** | **E1 C1** | **E1 C1** |
| **XIII** | **XIII** | **XIII** | ***CAT8*** | **E1 C1** | **U1 C1** |  | **E1 C1** | **E1 C1** | **E1 C1** | **E1 C1** |
| **XVI** | **XVI** | **XVI** | ***GAL4*** | **C1** | **U1 C1** |  | **E1 C1** | **C1** | **C1** | **C1** |
| **XVI** | **XVI** | **XVI** | ***JIP5*** | **E1 C1** | **U1 C1** |  | **E1 C1** | **E1** | **E1** | **E1** |
| **VIIItXV** | **VIIItXV** | **VIII** | ***CBP2*** | **E1 C1** | **U1 C1** |  | **E1 C1** | **E1 C1** | **E1 C1** | **E1 C1** |
| **VIIItXV** | **VIIItXV** | **XV** | ***ATF1*** | **E1 C1** | **U1 C1** |  | **E1 C1** | **E1 C1** | **E1 C1** | **E1 C1** |
| **XVtVIII** | **XVtVIII** | **XV** | ***RRI2*** | **E1 C1** | **U1 C1** |  | **E1 C1** | **E1 C1** | **E1 C1** | **E1 C1** |
| **XVtVIII** | **XVtVIII** | **VIII** | ***MNL1*** | **E1 C1** | **U1 C1** |  | **E1 C1** | **C1** | **E1 C1** | **E1 C1** |
| **VI** | **VItX** | **VI** | ***EPL1*** | **E1 C1** | **U1 C1** |  | **E1** | **E1** | **C1** | **E1** |
| **VI** | **VItX** | **VI** | ***GSY1*** | **E1 C1** | **U1 C1** |  | **E1** | **E1** | **U1 C1** | **E1** |
| **X** | **VItX** | **X** | ***PEX2*** | **E1 C1** | **C2 C1** |  | **E1 C1** | **E1 C1** | **E1 C1** | **E1 C1** |
| **X** | **XtVI** | **X** | ***CYR1*** | **E1 C1** | **U1 C1** |  | **E1 C1** | **E1 C1** | **E1 C1** | **E1 C1** |
| **IItIV** | **XIVtIItIV** | **IV** | ***EUG1*** | **E1 C1** | **U1 C1** |  | **E1** | **E1** | **E1** | **E1** |
| **IItIV** | **IVtIItII** | **II** | ***PKC1*** | **E1 C1** | **U1 C1** |  | **E1 C1** | **E1 C1** | **E1 C1** | **E1 C1** |
| **IVtII** | **IVtIItII** | **IV** | ***RPN4*** | **E1 C1** | **U1 C1** |  | **E1 C1** | **E1 C1** | **E1 C1** | **E1 C1** |
| **IVtII** | **IVtIItII** | **IV** | ***UGA3*** | **E1 C1** | **U1 C1** |  | **E1 C1** | **E1 C1** | **E1 C1** | **E1 C1** |
| **IVtII** | **IItIItXIV** | **II** | ***APM3*** | **E1 C1** | **U1 C1** |  | **E1** | **E1 C1** | **E1 C1** | **E1 C1** |
| **IVtII** | **IItIItXIV** | **II** | ***OPY1*** | **E1 C1** | **U1 C1** |  | **E1 C1** | **E1 C1** | **E1 C1** | **E1 C1** |
| **XIV** | **IItIItXIV** | **XIV** | ***EGT2*** | **E1 C1** | **U1 C1** |  | **E1** | **E1** | **U1 C1** | **E1 C1** |
| **XIV** | **XIVtIItIV** | **XIV** | ***BRE5*** | **E1 C1** | **U1 C1** |  | **E2 C1** | **E2** | **E2 C1** | **E2 C1** |
