# Supplementary material for: On the Complexity of the Saccharomyces bayanus Taxon: Hybridization and Potential Hybrid Speciation
Source: PLoS One. 2014 Apr 4;9(4):e93729. doi: 10.1371/journal.pone.0093729 (PMC3976317; doi:10.1371/journal.pone.0093729)
Supplement: File S1 — Contains the files: Figure S1 Chromosome composition and gene order in different Saccharomyces species. A- S. cerevisiae. B- S. eubayanus. C- S. uvarum. Figure S2 Phylogenetic analysis of the 5′ and 3′ regions of the mitochondrial COX2 gene. A- 5′ region. B- 3′ region. Table S1 Gene regions under restriction analysis and primers used for PCR amplification. Chromosome (Chr) positions of the genes correspond to S. cerevisiae, for other arrangements present in the other strains see Figure S1. Table S2 Composite restriction patterns deduced from the gene region sequences of the eubayanus -type alleles, present in the reference strains S. bayanus NBRC 1948, CECT 11186, CBS 424 or S. pastorianus Weihenstephan 34/70, the uvarum alleles exhibited by S. uvarum CBS 7001, and the cerevisiae -type alleles present in S. cerevisiae S288c. These composite patterns for each gene region have been named after the initial of the allele-type name followed by the order numeral 1. Chromosome (Chr) positions of the genes correspond to S. cerevisiae, for other arrangements present in the other strains see Figure S1. Table S3 Alternative restriction patterns exhibited by S. bayanus or S. uvarum strains differing by one or two restriction site gains/losses (indicated in bold) from those found in the reference strains. Table S4 Conformation of the S. uvarum strains for each gene region according to the composite restriction patterns exhibited. For a description of the composite restriction patterns, see Tables S2 and S3. Mitochondrial COX2 sequence haplotypes are described in Figure 2. Table S5 Conformation of the S. bayanus strains with eubayanus - and uvarum -type alleles according to the composite restriction patterns exhibited. For a description of the composite restriction patterns, see Tables S2 and S3. Mitochondrial COX2 sequence haplotypes are described in Figure 2. Table S6 Conformation of the S. pastorianus strains with eubayanus - cerevisiae - or uvarum -type alleles according to t [file pone.0093729.s001.zip › Figure S2.pptx]

## Slide 1
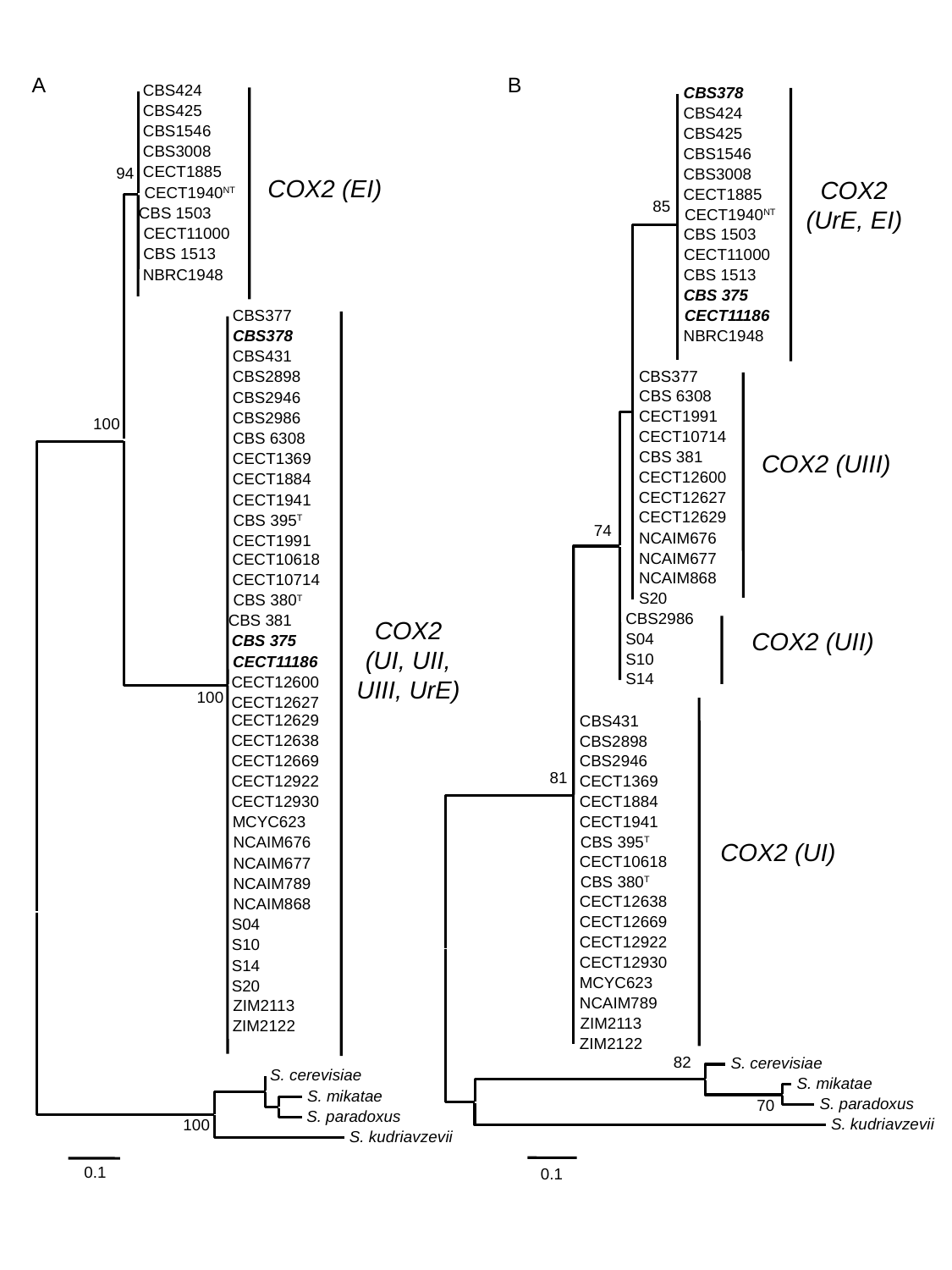

A
B
 CBS424
 CBS378
 CBS425
 CBS424
 CBS1546
 CBS425
 CBS3008
 CBS1546
 CECT1885
94
 CBS3008
COX2 (EI)
COX2 (UrE, EI)
 CECT1940NT
 CECT1885
85
CBS 1503
 CECT1940NT
 CECT11000
 CBS 1503
 CBS 1513
 CECT11000
 NBRC1948
 CBS 1513
 CBS 375
 CBS377
 CBS378
 CBS431
 CBS2898
 CBS2946
 CBS2986
 CBS 6308
 CECT1369
 CECT1884
 CECT1941
 CBS 395T
 CECT1991
 CECT10618
 CECT10714
 CBS 380T
CBS 381
 CBS 375
 CECT11186
 CECT12600
 CECT12627
 CECT12629
 CECT12638
 CECT12669
 CECT12922
 CECT12930
 MCYC623
 NCAIM676
 NCAIM677
 NCAIM789
 NCAIM868
 S04
 S10
 S14
 S20
 ZIM2113
 ZIM2122
 CECT11186
 NBRC1948
 CBS377
 CBS 6308
 CECT1991
100
 CECT10714
COX2 (UIII)
 CBS 381
 CECT12600
 CECT12627
 CECT12629
74
 NCAIM676
 NCAIM677
 NCAIM868
 S20
COX2
(UI, UII, UIII, UrE)
 CBS2986
COX2 (UII)
 S04
 S10
 S14
100
 CBS431
 CBS2898
 CBS2946
81
 CECT1369
 CECT1884
 CECT1941
COX2 (UI)
 CBS 395T
 CECT10618
 CBS 380T
 CECT12638
 CECT12669
 CECT12922
 CECT12930
 MCYC623
 NCAIM789
 ZIM2113
 ZIM2122
82
 S. cerevisiae
 S. cerevisiae
 S. mikatae
 S. mikatae
 S. paradoxus
70
 S. paradoxus
 S. kudriavzevii
100
 S. kudriavzevii
0.1
0.1
